# A new hybrid method to improve the ultra-short-term prediction of LOD

**DOI:** 10.1007/s00190-020-01354-y

**Published:** 2020-02-05

**Authors:** Sadegh Modiri, Santiago Belda, Mostafa Hoseini, Robert Heinkelmann, José M. Ferrándiz, Harald Schuh

**Affiliations:** 1 GFZ German Research Centre for Geosciences, Potsdam, Germany; 2 Institute for Geodesy and Geoinformation Science, Technische Universität Berlin, Berlin, Germany; 3 Image Processing Laboratory (IPL) - Laboratory of Earth Observation (LEO), University of Valencia, Valencia, Spain; 4 UAVAC, University of Alicante, Alicante, Spain; 5 Department of Civil and Environmental Engineering, Norwegian University of Science and Technology, Trondheim, Norway

**Keywords:** LOD, EOP, Copula-based analysis, Prediction

## Abstract

Accurate, short-term predictions of Earth orientation parameters (EOP) are needed for many real-time applications including precise tracking and navigation of interplanetary spacecraft, climate forecasting, and disaster prevention. Out of the EOP, the LOD (length of day), which represents the changes in the Earth’s rotation rate, is the most challenging to predict since it is largely affected by the torques associated with changes in atmospheric circulation. In this study, the combination of Copula-based analysis and singular spectrum analysis (SSA) method is introduced to improve the accuracy of the forecasted LOD. The procedure operates as follows: First, we derive the dependence structure between LOD and the *Z* component of the effective angular momentum (EAM) arising from atmospheric, hydrologic, and oceanic origins (AAM + HAM + OAM). Based on the fitted theoretical Copula, we then simulate LOD from the *Z* component of EAM data. Next, the difference between LOD time series and its Copula-based estimation is modeled using SSA. Multiple sets of short-term LOD prediction have been done based on the IERS 05 C04 time series to assess the capability of our hybrid model. The results illustrate that the proposed method can efficiently predict LOD.

## Introduction

Earth orientation parameters (EOP) are a collection of parameters that describe irregularities in the rotation of the Earth. EOP are classified into three groups: polar motion (PM) given by the *x*, *y*,  parameters; diurnal rotation (e.g., ERA = Earth rotation angle, or UT1-UTC); and precession–nutation (PN) pair, which give the orientation of the conventional Celestial Intermediate Pole (CIP) in the geocentric celestial reference frame. The EOP can be observed with modern high-precision space geodetic techniques, such as very long baseline interferometry (VLBI), satellite laser ranging (SLR), and global positioning system (GPS) (Tapley et al. 1985; Lichten et al. 1992; Schuh and Schmitz-Hübsch 2000). Real-time EOP estimation is needed for many applications including precise tracking and navigation of interplanetary spacecraft, climate forecasting, and disaster prevention. However, the complexity and time-consuming in data processing always lead to time delays. Consequently, the prediction of EOP from past observed data or combining with the geophysical phenomena is of great scientific and practical importance. In addition to the five EOP, the length of day (LOD) is used to model the variations in the Earth’s rotation rate. LOD is the difference between the duration of the day measured by space geodesy and nominal day of 86,400 s duration and defined as: $\mathrm{LOD}=-\frac{\mathrm{d}({\mathrm{UT}}1{{\text {-}}}{\mathrm{UTC}})}{\mathrm{d}t}$ (Freedman et al. 1994). LOD is changing due to gravitational effects from external bodies and geophysical processes occurring in different Earth layers. Consequently, its knowledge is essential for various applications related to reference frame determination and metrology, such as interplanetary navigation and space geodesy orbitography (i.e., precise orbit determination) because of its coupling with the orbit node. However, the LOD prediction is extremely difficult due to extreme events such as El Niño which demonstrated themselves in the LOD signals (Holton and Dmowska 1989; Gross et al. 1996).

Several techniques have been developed to improve the accuracy of LOD prediction. These algorithms could be classified into two groups: first, the methods that use the information within the LOD time series, e.g., auto-covariance (AC) (Kosek et al. 1998; Kosek 2002), wavelet decomposition (Akyilmaz et al. 2011), or neural network (Schuh et al. 2002; Liao et al. 2012; Lei et al. 2015, 2017). Besides, this group includes the hybrid methods using the combination of least squares (LS) and auto-regressive (AR), auto-regressive moving average (ARMA), auto-covariance, and neural network (Kosek et al. 2005; Xu and Zhou 2015; Wu et al. 2019). In the second group, we cast the methods that take into account the axial component of effective angular momentum ($\mathrm{EAM}_{Z}$) (Freedman et al. 1994; Gross et al. 1998; Johnson et al. 2005; Niedzielski and Kosek 2008; Kosek 2012; Nastula et al. 2012; Dill et al. 2019). Freedman et al. (1994) showed that the use of atmospheric angular momentum (AAM) wind terms in the Kalman filter technique to predict LOD variations improved near-term predictions. Johnson et al. (2005) used UT1-like observations determined by AAM in the UT1-UTC combination solution to predict UT1 which showed a significant reduction in the prediction errors when compared with the previous prediction method (McCarthy and Luzum 1991). Also, Dill et al. (2019) used 6 days long predicted EAM values for the PM and UT1-UTC prediction using LS extrapolation and AR model. The Earth orientation parameters prediction comparison campaign (EOP PCC) took place within 2005–2009, and it was organized to assess the various prediction techniques under the same conditions and rules. One of the main results of the EOP PCC was that there is not a specific method preferred for all EOP and all prediction intervals (ultra-short term and long term). Also, as the EOP prediction accuracy benefits from AAM forecast data, EOP PCC recommended paying more attention to the analysis and prediction of atmospheric angular momentum (AAM), continental hydrology angular momentum (HAM), and ocean angular momentum (OAM) (Kalarus et al. 2010). Therefore, a new prediction method is required to fully capture the dependence structure between AAM, HAM, OAM, and EOP. Although there are approaches to quantify the dynamical relation between the geophysical fluids (the atmosphere, the ocean, and the land hydrology) and the LOD variation (Gross 2015), we ignore them in our work as we exactly want to assess this relation directly independent of theoretical implications through comparing the LOD and the effective angular momentum function z-component by the Copula method. In this paper, we introduce an algorithm to improve the LOD prediction for reaching the accuracy goals pursued by the Global Geodetic Observing System (GGOS) of the International Association of Geodesy (IAG), i.e., 1 mm accuracy and 0.1 mm/year stability on global scales in terms of the International Terrestrial Reference Frame (ITRF) defining parameters (Plag and Pearlman 2009). We explored the combination of Copula-based analysis and singular spectrum analysis (SSA) to predict LOD. In Modiri et al. (2018), we applied the combination of SSA and Copula for the first time as a novel deterministic-stochastic tool for PM prediction. In this method, deterministic part is estimated by SSA, whereas Copula is used for modeling the stochastic part. The results indicated that the proposed approach can efficiently predict PM. Moreover, the improvement in PM prediction accuracy up to 365 days in the future is found to be 40% on average compared to the current PM prediction data from the International Earth Rotation and Reference Systems Service (IERS) Rapid Service/Prediction Center (RS/PC), hosted by the US Naval Observatory (USNO) (Petit and Luzum 2010). The Copula method contains both linear and nonlinear dependence structures between variables, and it is a powerful tool for dealing with multi-dimensional data and for modeling the relationship between parameters (Joe 1997). SSA is a subspace-based technique which makes use of empirical functions derived from the data to model the time series in a pre-specified level of details. It can be used for trend extraction and extrapolation (Alexandrov 2009; Modiri et al. 2018), periodicity detection, seasonal adjustment, smoothing, noise reduction (Golyandina et al. 2001; Ghil et al. 2002) as well as for change point detection (Moskvina and Zhigljavsky 2003; Hoseini et al. 2020). First, we derive the dependence structure between LOD and $\mathrm{EAM}_{Z}$. Based on the fitted theoretical Copula, we then simulate LOD from the $\mathrm{EAM}_{Z}$ data. Next, the difference between LOD time series and its Copula-based estimation is modeled using SSA. After that, the LOD will be computed from predicted $\mathrm{EAM}_{Z}$. Finally, the difference will be predicted and will be added to LOD predicted by Copula. Multiple sets of ultra-short-term (10 days) LOD prediction have been made based on IERS 05 C04 time series to assess the capability of our hybrid model. We consider the same conditions as EOP PCC to show the effectiveness of the presented method. We compare the prediction results with those of existing techniques of EOP PCC, and the results evidence that the proposed approach can efficiently predict LOD.

## Methods

The combination of stochastic and deterministic methods is used for LOD prediction. The Copula-based analysis technique aims to estimate the models for capturing the dependence structure between observed LOD and the $\mathrm{EAM}_{Z}$. Also, SSA is used as a deterministic technique to obtain stochastic residuals (the difference between the observed data and the Copula generated data). Finally, let us remark that we used the IERS EOP time series available at the time of the EOP PCC so that our results could be easily compared to the former analyses. In the following section, the theoretical background of Copula theory and SSA is briefly sketched.

### Copula-based analysis

The Copula approach exploits linear and nonlinear dependency between variables. Copula is a flexible tool offering an enormous improvement in capturing the real correlation pattern. This technique provides the grounds for dealing with multi-dimensional data and modeling the relation between parameters based on the marginal distribution functions of the variables (Embrechts et al. 2002).

Copula appeared in the mathematics context for the first time by Sklar (1959). Sklar’s theorem indicates that a Copula function *C* connects a given multivariate distribution function with its univariate marginal. For bivariate distribution, there is a bivariate Copula *C* which models the joint cumulative probability distribution function of two variables *X* and *Y* based on the marginal cumulative distribution functions $F_{X} (x)$ and $F_{Y}(y)$.

1 $$\begin{aligned} \begin{aligned} P(X\le x, Y\le y)= F_{X,Y}(x, y)&=C(F_X(x), F_Y(y))\\&= C(u, v) \end{aligned} \end{aligned}$$

where *C* describes the joint distribution function $F_{X,Y}(x, y)$. The variables *u* and *v* are transformations of *X* and *Y* to uniform distribution, respectively. The Copula is unique when the marginals are continuing functions. As the Copula is a reflection of the dependence structure itself, its construction is reduced to the study of the relationship between the variables, giving freedom for the choice of the univariate marginal distribution. Further information about Copula can be found, e.g., in Joe (1997) and Nelsen (2006). For many years, the Copula method has been used for modeling the dependence structure between random variables in different types of studies, such as Economics (Rachev and Mittnik 2000; Patton 2006, 2009), Biomedicine (Wang and Wells 2000; Escarela and Carriere 2003), Hydrology (Bárdossy and Li 2008; Bárdossy and Pegram 2009; Verhoest et al. 2015), Meteorology (Laux et al. 2011; Vogl et al. 2012), Hydro-geodesy, and Geodesy (Modiri et al. 2015, 2018). Six different bivariate Copula families are used in this research: the Archimedean 12, Archimedean 14, Clayton, Frank, Gumbel, and Joe. Further information about mathematical details on these families can be found in Appendix A, Nelsen (2006), or in Salvadori and De Michele (2007).

### Singular Spectrum Analysis

SSA is a time series analysis tool which can be used to retrieve robust components of a dataset aiming to provide an easier to interpret picture of complex observations. The method diagonalizes a lag-covariance matrix concerning a basis of orthogonal eigenvectors and computes the corresponding eigenvalues (Groth and Ghil 2015). SSA is able to reveal useful information about hidden underlying processes of a time series. Within its four steps, SSA groups correlated information in a time series and offers the opportunity of reproducing new versions of the time series based on their different characteristics [see Appendix B, Golyandina et al. (2001), and Ghil et al. (2002), for more details].

### Error analysis

The mean absolute error (MAE) is used in order to evaluate the prediction accuracy. The MAE is calculated for the *k*th day in the future as follows:

2 $$\begin{aligned} \mathrm{MAE} = \frac{1}{k}\sum _{i=1}^k(|P_{i}-O_{i}|) \end{aligned}$$

where $P_{i}$ is the predicted value of the *i*th prediction day, $O_{i}$ is the corresponding observed value, and *k* is the total prediction number.

## Calculation and analysis

### Data description

#### Length of day (LOD)

Daily time series of LOD in this contribution are from IERS EOP 05 C04 series (Gambis 2004). The LOD time series are available from (http://hpiers.obspm.fr/eop-pc) and span the time interval from 1996 to 2008.

**Fig. 1:**
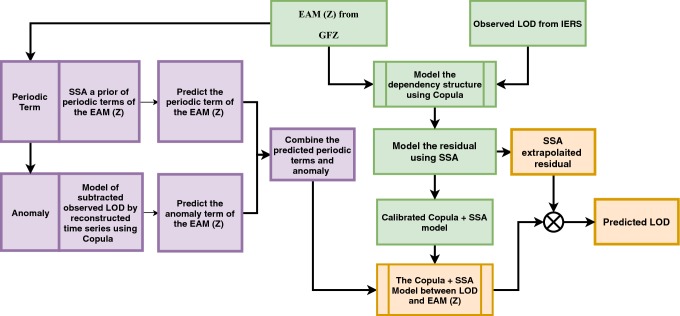
Scheme of the prediction algorithm (Copula + SSA model). The Copula-based joint distribution function between LOD and $\mathrm{EAM}_{Z}$ (Calibration step) is shown in green. $\mathrm{EAM}_{Z}$ prediction is shown in purple. The prediction of LOD using the calibrated model (final step) is illustrated by orange

#### Effective angular momentum (EAM) functions

EAM functions in both mass and motion terms explain the non-tidal geophysical excitation of the Earth’s rotation due to mass redistribution in atmospheric and terrestrial hydrosphere, and in the oceans. The EAM data consist of three main components *X*, *Y*, and *Z*. The *X* and *Y* components are associated with the excitation of polar motion, whereas the *Z* component is responsible for the excitation of LOD variation (Salstein 1993; Dobslaw and Thomas 2005; Dill 2008; Jungclaus et al. 2013; Dobslaw 2016). The EAM functions are dimensionless with the sampling of 1 day and are provided by the Earth System Modeling group at Deutsches GeoForschungsZentrum Potsdam (ESMGFZ) (Dobslaw and Dill 2018). The EAM time series are available from: (ftp://ig2-dmz.gfz-potsdam.de/EAM/).

### Data processing and analysis

**Fig. 2:**
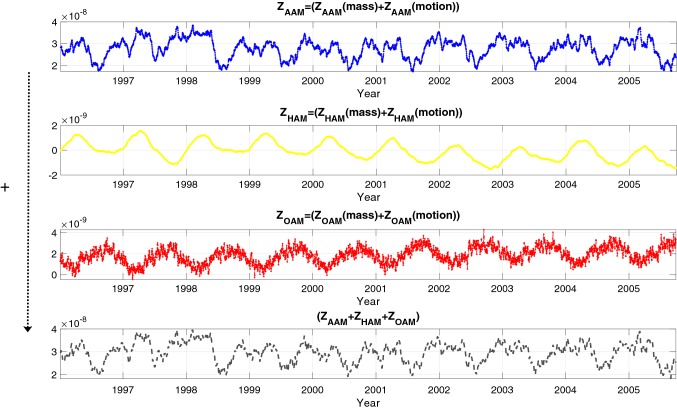
The $\mathrm{EAM}_{Z}$ being the sum of mass and motion terms of AAM, HAM, and OAM ($Z_\mathrm{AAM}+Z_\mathrm{HAM}+Z_\mathrm{OAM}$)

**Fig. 3:**
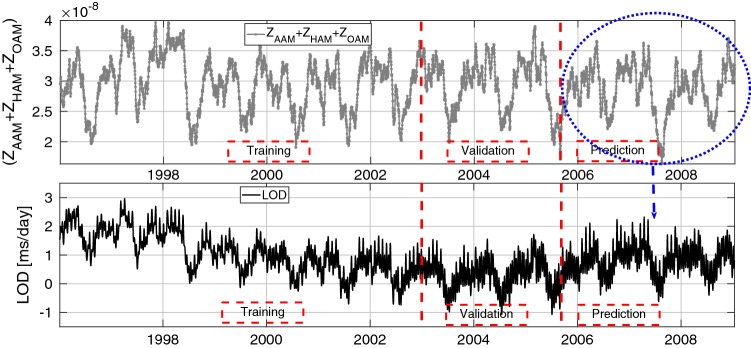
Time series of LOD and ($Z_\mathrm{AAM}+Z_\mathrm{HAM}+Z_\mathrm{OAM}$) between 1996 and 2008. The time series is divided into three parts: training part (1996–2003), validation (2003–2005), and prediction (2005–2008)

In this paper, we defined an algorithm for LOD prediction as shown in Fig. 1. It is important to note that LOD can be decomposed into several components (e.g., variations related to zonal components of solid Earth tides and ocean tides, atmospheric circulation, internal effects, and transfer of angular momentum to the Moon orbital motion). Taking into account that the accuracy of the different models may be not homogeneous, we decide to include in the modeling of this study the total variation in LOD. We are aware that it may be more challenging for testing the method performance that relying partially in previous models for certain components, but we preferred not to remove too many difficulties when testing the method as a mean to get more insight into its capabilities. Having said that, the methodology is structured as follows. We analyze the $\mathrm{EAM}_{Z}$ which is the sum of mass and motion terms of AAM, HAM, and OAM (see Fig. 2). The dependence structure between observed LOD and $\mathrm{EAM}_{Z}$ is captured and modeled by using Copula-based analysis. The difference between the observed LOD and Copula LOD estimated data is modeled using the SSA method. After that, the $\mathrm{EAM}_{Z}$ is predicted as described in detail in Modiri et al. (2018). The prediction algorithm is demonstrated through the following steps:

1. Copula-based joint distribution function of LOD and $\mathrm{EAM}_{Z}$
  Model the dependency structure between LOD and $\mathrm{EAM}_{Z}$
  Model the periodic residual using SSA
  Calibrate the Copula + SSA model
2. $$\mathrm{EAM}_{Z}$$ prediction
  SSA periodic terms estimation
  Copula anomaly modeling
3. LOD prediction using the calibrated Copula + SSA model
  Sample random data from the conditional Copula;

as it is shown in Fig. 3, the training interval is between 1996 and 2003.

#### Copula-based joint distribution function of LOD and $EAM_Z$

**Fig. 4:**
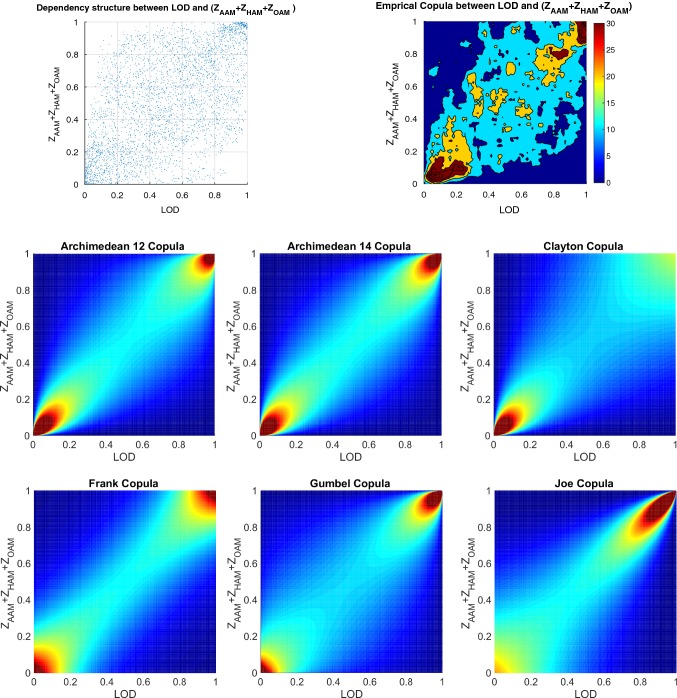
Scatter plot of LOD and ($\mathrm{EAM}_{Z}$) and its empirical Copula (upper panel). The fitted Archimedean 12 ($\theta =1.30$), Archimedean 14 ($\theta =1.53$), and Clayton ($\theta =1.31$) Copula (middle panel), Frank ($\theta =5.10$), Gumbel ($\theta =1.69$), and Joe ($\theta =2.92$) Copula (lower panel) between 1996 and 2003 in the rank space [0 1]

In this study, the training dataset which ranges from 1996 to 2003 is used to fit a Copula-based joint distribution function; both $\mathrm{EAM}_{Z}$ and LOD are transformed to uniformly distributed values between (0, 1) interval through their empirical cumulative distribution function. Thereafter, the dependence structure between the $\mathrm{EAM}_{Z}$ and LOD is investigated. First, the empirical Copula is estimated using the Eq. 3. As it can be seen in Fig. 4, there is a scatter plot (upper panel) of LOD and $\mathrm{EAM}_{Z}$, and it shows approximately a linear dependence structure with both upper and lower heavy tail which can be modeled by using the Archimedean Copula. Therefore, the theoretical bivariate Archimedean Copula functions with their estimated parameters are fitted to the estimated empirical Copula. The LOD data are sampled based on the Copula and the empirical marginal distribution of $\mathrm{EAM}_{Z}$. Next, the residuals of the generated LOD by Copula are estimated using SSA. After that, the ($Z_\mathrm{AAM}+Z_\mathrm{HAM}+Z_\mathrm{OAM}$) data between 2003 and 2005 are used for the validation of LOD prediction (calibration step). Here, the predicted $\mathrm{EAM}_{Z}$ are used for the LOD prediction in the time interval between 2005 and 2008.

**Fig. 5:**
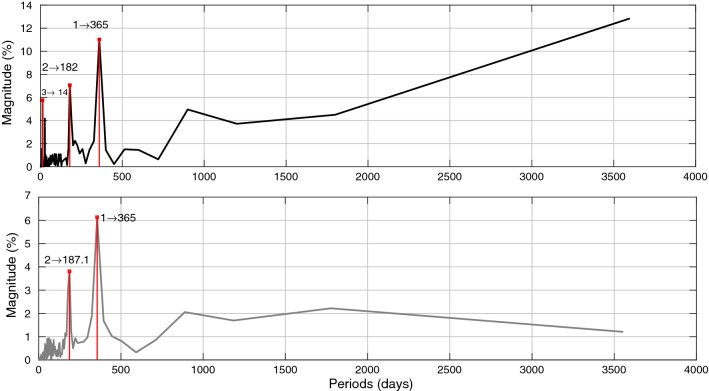
Spectral analysis of the LOD (up), $Z_\mathrm{AAM}+Z_\mathrm{HAM}+Z_\mathrm{OAM}$ (down) using fast Fourier transform (FFT)

**Fig. 6:**
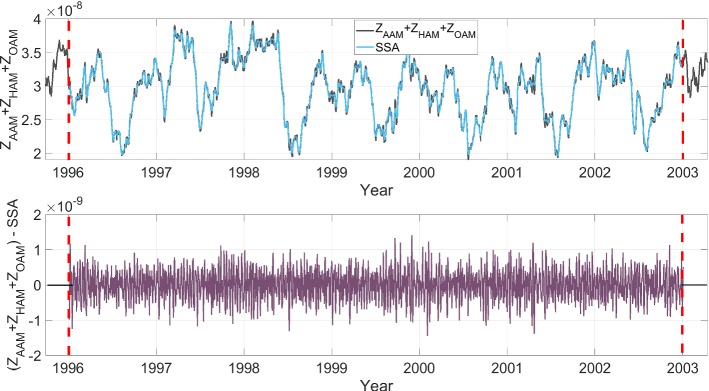
The original time series and the reconstructed time series (upper panel), and the difference between the original and reconstructed time series (lower panel) for ($Z_\mathrm{AAM}+Z_\mathrm{HAM}+Z_\mathrm{OAM}$)

#### $\mathrm{EAM}_{Z}$ prediction

**Fig. 7:**
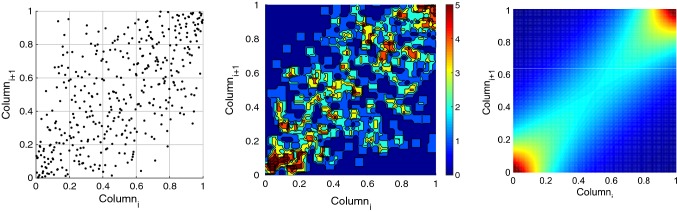
Scatter plot (left) two adjacent columns in the residual matrix. The empirical Copula (middle) is estimated based on the dependency structure of two columns. The Frank Copula with $\theta = 4.79$ is fitted to the empirical Copula (right)

**Fig. 8:**
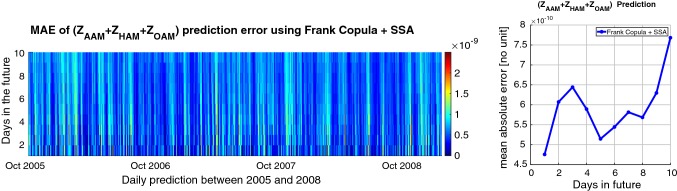
MAE of ($Z_\mathrm{AAM}+Z_\mathrm{HAM}+Z_\mathrm{OAM}$) prediction between 2005 and 2008 (left). The MAE of ($Z_\mathrm{AAM}+Z_\mathrm{HAM}+Z_\mathrm{OAM}$) prediction between 2005 and 2008 (right)

For this step, we defined an algorithm for $\mathrm{EAM}_{Z}$ prediction as shown in Fig. 1. The ($Z_\mathrm{AAM}+Z_\mathrm{HAM}+Z_\mathrm{OAM}$) time series can be split up into two parts. The first part is dealing with periodic effects such as annual and semiannual variations due to the spectral analysis of $\mathrm{EAM}_{Z}$ (illustrated in Figure 5). The SSA models the periodic terms of the ($Z_\mathrm{AAM}+Z_\mathrm{HAM}+Z_\mathrm{OAM}$) (see Fig. 6). Then, the difference between the observed $\mathrm{EAM}_{Z}$ and SSA estimated data is modeled by using the Copula-based analysis method. First, the window length ($L=365$ days) is selected considering the main periodicity (see Figure 5). After that, the number of singular vectors for reconstruction of the $\mathrm{EAM}_{Z}$ time series is determined. The trajectory matrix is constructed by having the window length and number of singular vectors. The cyan curve depicts the SSA-reconstructed $\mathrm{EAM}_{Z}$ time series. The periodic terms of $\mathrm{EAM}_{Z}$ are extrapolated using the SSA as a priori model. The difference between the values predicted by SSA and the predictions based on the $\mathrm{EAM}_{Z}$ is called $\mathrm{EAM}_{Z}$ anomaly and has a stochastic nature. This anomaly part is predicted using the Copula-based model. The anomaly part is displayed in Fig. 6 (lower panel). This part is formed with the same window length *L*. The dependence structure between the $\mathrm{column}_{i}$ and $\mathrm{column}_{i+1}$ in the residual matrix is investigated. As can be seen in Fig. 7, the scatter plot illustrates a linear dependence between the two adjacent columns which are modeled by Archimedean Copula. Then, the empirical Copula is determined using Eq. 3. The next step is fitting a bivariate Archimedean Copula. In this study, Frank Copula is selected for predicting the $\mathrm{EAM}_{Z}$ anomaly due to its ability to capture the linear dependence structure. Finally, the Copula-based predicted anomalies are added to the previously described deterministic part. Here, we utilized seven years of $\mathrm{EAM}_{Z}$ time series from September 1998 to September 2005 for the 10 days ahead forecasting during the interval between October 2005 and 2008, i.e., the same interval as it has been used for the EOP PCC. As can be seen in Fig. 8, the MAE of the $\mathrm{EAM}_{Z}$ prediction is up to the 0.76 for the next days which is two orders of magnitude smaller than the $\mathrm{EAM}_{Z}$ magnitude. In Fig. 7, the scatter plot shows a linear relationship between $\mathrm{column}_{i}$ and $\mathrm{column}_{i+1}$. Then, the corresponding empirical Copula is estimated. Finally, the anomalies are added to the SSA-forecasted time series.

### LOD prediction from predicted $\mathrm{EAM}_{Z}$ using the calibrated Copula + SSA model

The predicted $\mathrm{EAM}_{Z}$ dataset from 2005 to 2008 is employed as input time series for the calibrated model. As can be seen in Fig. 1, the periodic terms in the residual part are predicted using SSA extrapolation as well. Finally, the Copula-based predicted data are added to the SSA-forecasted residual. To asses the proposed method, the results are compared with the EOP PCC solutions.

**Fig. 9:**
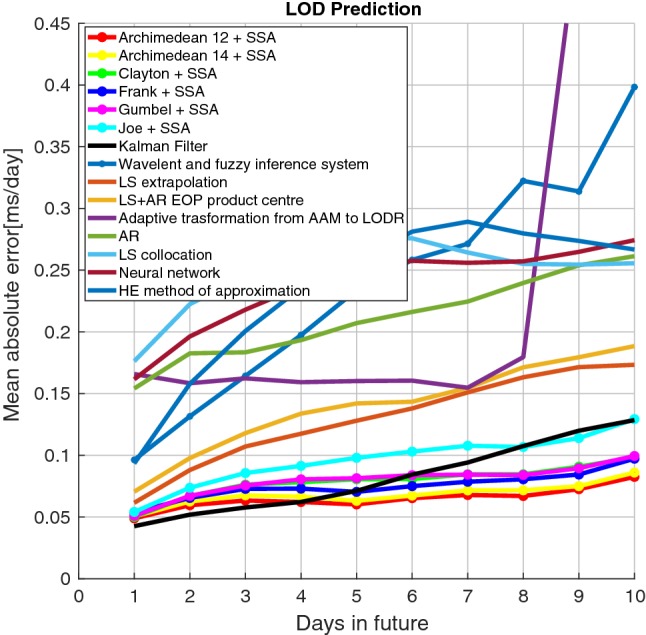
Mean absolute errors of the predicted LOD using Archimedean 12 + SSA, Archimedean 14 + SSA, Clayton Copula + SSA, Gumbel Copula + SSA, Frank Copula + SSA, Joe Copula + SSA, and EOP PCC results

**Table 1:** Comparison of Copula + SSA prediction and EOP PCC prediction errors (unit: ms/day)

Prediction day	1	2	3	4	5	6	7	8	9	10
Archi 12 + SSA	0.047	0.060	0.063	0.063	0.059	0.064	0.066	0.061	0.072	0.083
Archi 14 + SSA	0.050	0.065	0.066	0.068	0.064	0.067	0.072	0.064	0.073	0.082
Clayton + SSA	0.051	0.067	0.079	0.085	0.079	0.078	0.084	0.078	0.086	0.093
Gumbel + SSA	0.052	0.065	0.073	0.076	0.070	0.076	0.085	0.080	0.082	0.094
Frank + SSA	0.047	0.062	0.070	0.086	0.083	0.081	0.084	0.085	0.092	0.097
Joe + SSA	0.052	0.079	0.081	0.088	0.097	0.102	0.116	0.111	0.120	0.121
Kalman filter	0.042	0.051	0.057	0.062	0.071	0.084	0.094	0.107	0.119	0.128
wavelet	0.096	0.131	0.164	0.197	0.233	0.258	0.271	0.322	0.313	0.398
LSE	0.061	0.088	0.107	0.117	0.128	0.138	0.151	0.163	0.171	0.173
LS+AR EOP PC	0.070	0.097	0.118	0.133	0.142	0.143	0.154	0.171	0.179	0.188
Adaptive transform	0.165	0.158	0.162	0.159	0.160	0.160	0.154	0.179	0.528	0.593
AR	0.154	0.182	0.183	0.193	0.207	0.216	0.224	0.239	0.253	0.261
LSC	0.176	0.222	0.245	0.266	0.276	0.275	0.264	0.255	0.254	0.255
NN	0.161	0.196	0.218	0.237	0.250	0.257	0.256	0.257	0.264	0.274
HE	0.093	0.157	0.200	0.235	0.257	0.281	0.289	0.279	0.273	0.266

## Discussion of the results

**Fig. 10:**
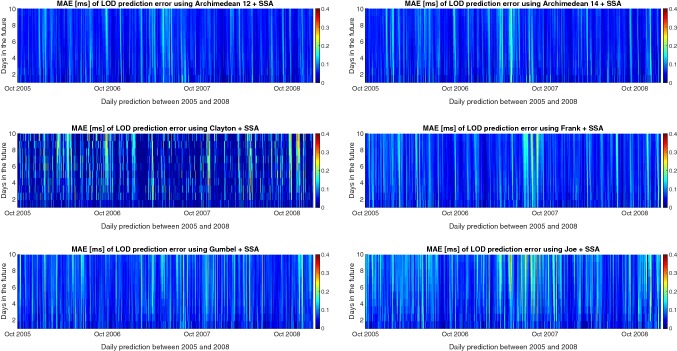
Absolute errors of the predicted LOD using Archimedean 12 + SSA, Archimedean 14 + SSA, Clayton Copula + SSA, Gumbel Copula + SSA, Frank Copula + SSA, Joe Copula + SSA

In this paper, a hybrid LOD prediction method Copula+SSA has been tested. The proposed combination method is examined based on the hind-cast experiments using the data from the past, i.e., the LOD data are predicted using the same time span (2005–2008) as the EOP PCC. Figure 9 shows the MAE of ultra-short-term prediction. The MAE of our hybrid Copula + SSA models indicates fewer errors compared to the EOP PCC solutions. However, the Kalman filter with forecasted AAM shows a comparable performance with our proposed model with smaller MAE for the first 4 days in the future. Table 1 presents the MAE of Copula + SSA and EOP PCC results in numbers. Adoptive transform, AR, LS collocation, and NN show errors of more than $0.1\,\mathrm{ms/day}$ for the first day of prediction. Also, the MAE of the wavelet, LS extrapolation LS + AR, and He approaches reach more than 0.1 ms/day after 2 or 3 days in the future. From all contributions to the EOP PCC solution, Kalman filter provides the best accuracy. However, the MAE of the Kalman filter gets larger than $0.1\,\mathrm{ms/day}$ after 7 days. All Copula + SSA models show MAE smaller than 0.1 ms/day over 10-day prediction, except smaller Joe Copula + SSA. Figure 10 presents the MAE of 10-day-ahead prediction between 2005 and 2008 for all six hybrid models to understand better the prediction performance and its causes. Different features can be seen, and there are some common patterns such as high errors from the beginning of 2007 which might be caused by the El Niño effect or certain geomagnetic jerk events as pointed out by Shirai et al. (2005) or Malkin (2013). As NOAA National Centers for Environmental Information, State of the Climate reported, the El Niño warm event had a peak in December 2006 and started to dissipate during January 2007. Thus, the equatorial Pacific sea-surface temperature (SST) anomalies decreased during the first two months of 2007, eventually declining to near average by the end of March 2007. Kalarus et al. (2010) suggested benefiting from the prediction of the AAM, OAM, and HAM. Therefore, our better prediction performance may be due to considering both mass and motion terms of $\mathrm{EAM}_{Z}$ for modeling the dependence structure between LOD and $\mathrm{EAM}_{Z}$. It is important to note that the Copula + SSA Archimedean 12 and 14 Copula provide significantly smaller errors than the other methods. On the other hand, the Joe Copula exhibits slightly larger errors than the two aforesaid models. This may have been caused by Archimedean 12 and 14 Copula’s ability to capture the upper and lower heavy tail dependence structure.

## Summary and Conclusion

LOD represents the variation in Earth’s rotation rate which is most difficult to predict, because of the occurrence of extreme events in the LOD signal. In this paper, we introduce several approaches based on Copulas which were applied to bivariate frequency analysis. Using Copula is promising since it allows to take into account a wide range of correlation, frequently observed in time series. The presented work here is aimed at the possibility of utilizing the $\mathrm{EAM}_{Z}$ data to predict LOD data due to the existing relationship between them. In order to study this relationship, two datasets were compared: the observed LOD from IERS EOP C05 and $\mathrm{EAM}_{Z}$ derived from GFZ. The comparison with results of other methods indicates that the Copula + SSA can efficiently and precisely predict the LOD parameter at ultra-short term. All of our methods introduced here provide comparable error with the existing methods used for their evaluation in the time interval considered of up to 10 days. Besides, it is clearly demonstrated that the predicted AAM, HAM, OAM time series as additional input information can improve the LOD prediction. Among the analyzed combinations, the Archimedean 12 + SSA and Archimedean 14 + SSA show the most sophisticated performance with low errors. As the Kalman filter prediction provides better results within the first 3 to 4 days, we will investigate this topic further in future in order to find out how the EAM functions can deliver even better LOD predictions. The EOP PCC proved once more to be very useful. As long as the data are still available for post-processing, new methods can adequately be compared in a consistent way to the methods applied in the past.

## Data Availability

The data that support the findings of this study are available from the corresponding author upon reasonable request.

## A Copula-based analysis

### A.1 Empirical Copula

The empirical Copula approximates the unknown theoretical Copula distribution. The empirical Copula is purely based on the data, and it is defined in the rank space as follows (Genest and Rivest 1993; Genest and Favre 2007; Laux et al. 2011):

3 $$\begin{aligned} C_\mathrm{e}(u,v)=\frac{1}{n} \sum _{i=1}^n \mathbf 1 \left( \frac{r_i}{n+1} \le u,\frac{s_i}{n+1} \le v \right) \end{aligned}$$

where

- $(r_1), (r_2) \ldots , (r_n)$ denote the pairs of ranks of the variable $(x_1),(x_2), \ldots , (x_n)$,
- $(s_1), (s_2) \ldots , (s_n)$ denote the pairs of ranks of the variable $(y_1),(y_2), \ldots , (y_n)$,
- *n* is the length of the data vector,
- **1**(...) is the indicator function. If the condition is true, the indicator function is equal to 1. Otherwise, the indicator function is equal to 0.

### A.2 Archimedean Copula

Some Copulas can be estimated directly with the simple form. They are named Archimedean Copulas. An Archimedean Copula can be described in the following form:

4 $$\begin{aligned} C(u, v)= \phi ^{-1}\lbrace \phi (u)+\phi (v),\theta \rbrace \end{aligned}$$

where $\theta$ is the Copula parameter and the function $\phi$ is the generator of the Copula with the following characteristics (Nelsen 2006):

**Table 2:** Six ordinary families of Archimedean Copulas (Archimedean 12, Archimedean 14, Clayton, Frank, Gumbel, and Joe Copula) and generator, parameter space, and their formula

Family	Generator	Parameter	Formula
Archimedean 12	$\phi ^{Arch12}(t)= (\frac{1}{t}-1)^{\theta }$	$1 \le \theta$	$C_\theta (u,v)= (1+[(u^{-1}-1)^{\theta }+(v^{-1}-1)^{\theta }]^{(\frac{1}{\theta })})^{-1}$
Archimedean 14	$\phi ^{Arch14}(t)= (t^{\frac{-1}{\theta }}-1)^{\theta }$	$1 \le \theta$	$C_\theta (u,v)= (1+[(u^{\frac{-1}{\theta }}-1)^{\theta }+(v^{\frac{-1}{\theta }}-1)^{\theta }]^{\frac{1}{\theta }})^{-\theta }$
Clayton	$\phi ^{Cl}(x)=\frac{1}{\theta }(t^{-\theta }-1)$	$-1 \le \theta$	$C_\theta (u,v)= \max [(u^{-\theta }+v^{-\theta }-1),0]^{-\frac{1}{\theta }}$
Frank	$\phi ^{Fr}(t)=-\ln \lbrace \frac{e^{-\theta t}-1}{e^{-\theta }-1}\rbrace$	$-\infty< \theta <\infty$	$C_\theta (u,v)= \frac{1}{\theta }\ln (1+ \frac{(e^{-\theta u}-1)(e^{-\theta v})}{e^{-\theta }-1})$
Gumbel	$\phi ^{Gu}(t)=(-\ln t)^\theta$	$1 \le \theta$	$C_\theta (u,v)= e^{-((-\ln (u)^\theta )+(-\ln (v)^\theta ))^{\frac{1}{\theta }}}$
Joe	$\phi ^{Joe}(t)=-\ln [1-(1-t)^\theta ]$	$1 \le \theta$	$C_\theta (u,v)= 1- [ (1-u)^\theta + (1-v)^\theta - ( (1-u)^\theta (1-v)^\theta ))]^\frac{1}{\theta }$

$\theta$ is the parameter of the Copula called the dependence parameter, which measures the dependence between the marginal (Nelsen 2006)

- for all $u \in (0,1), \phi (u) < 0$, $\phi$ is decreasing and convex (Table 2),
- $$ \phi (1)=0$$,

and $\phi ^{-1}$ is defined by

5 $$\begin{aligned} \phi ^{-1}(t)= {\left\{ \begin{array}{ll} \phi ^{-1}(t;\theta ),&{} \text {if }\, 0\le t \le \phi (0)\\ 0, &{} \text {if }\, \phi (0)\le t \le \infty \end{array}\right. } \end{aligned}$$

#### A.2.1 The relationship between Copula parameter $\theta$ and classical dependence parameter

There is a functional relationship between classical dependence parameters such as Kendall $\tau$ and Copula parameters. For one-parametric Copulas, the functional relationship between the Kendall $\tau$ Copula functions, namely:

6 $$\begin{aligned} \tau = 4 \int _{0}^1 \int _{0}^1 C_{\theta } (u,v) dC_{\theta } (u,v) -1, \end{aligned}$$

Equation 7 can be used to estimate the Copula parameter. Kendal $\tau$ for Archimedean Copula with the generator $\varPhi (t)$ is shown:

7 $$\begin{aligned} \tau =1+4\int _{0}^1 \frac{\varPhi (t)}{\varPhi (t)^{'}} dt. \end{aligned}$$

Thus, the relations between the Kendall $\tau$ and the Archimedean Copula parameters are illustrated in Table 3. Here D is Debye functions.

$$\begin{aligned} D_{k}(k)=\frac{k}{x^{k}} \int _{t=0}^{x} \frac{t}{e^{t}-1} dt \end{aligned}$$

**Table 3:** The link between Archimedean Copula parameter $\theta$ and Kendall $\tau$ (Cherubini et al. 2004)

Family	$\tau$
Archimedean 12	$\frac{\theta - \frac{2}{3}}{\theta }$
Archimedean 14	$\frac{2\theta -1}{2\theta +1}$
Clayton	$\frac{\theta }{\theta + 2 }$
Frank	$1-\frac{4}{\theta }[1-D_{1}(\theta )]^{*}$
Gumbel	$\frac{\theta -1}{\theta }$
Joe	$1-\frac{4}{\theta } D_{1}(\theta )$

* $D_{k}(x)$ is the Debye function for any positive integer k

### A.3 Simulating from Copula-based conditional random data

This subsection provides the essential steps for data simulation using Copula-based conditional random data. The following steps are taken to fit the proper theoretical Copula function and simulation data (Laux et al. 2011; Vogl et al. 2012; Modiri et al. 2018).

1. Independent identical distribution (iid)-transformation of input time series.
2. Compute the marginal distributions $$F_X (x)$$ and $$ F_Y (y)$$ of the input data *x* and *y*.
3. Transform data to rank space using the estimated marginal distributions of data with $u_i$ and $v_i$ in rank space.
4. Compute the empirical Copula to the dependence structure of random variables using the rank-transformed data.
5. Fit a theoretical Copula function $C_\theta (u,v)$.
6. Compute the conditional Copula function.
7. Sample random data from the conditional Copula cumulative distribution function (CDF).
8. Transfer the sample back to the data space using the inverse marginal.

## B Singular Spectrum Analysis

In the first step which is called embedding, we form a trajectory matrix ($\mathbf{X}$) by moving a window of length *L* over the elements of the time series ($f_i$), i.e.,

8 $$\begin{aligned} \mathbf{X}= & {} \begin{bmatrix} f_{1} &{} f_{2} &{} f_{3} &{} \dots &{} f_{K} \\ f_{2} &{} f_{3} &{} f_{4} &{} \dots &{} f_{K+1} \\ f_{3} &{} f_{4} &{} f_{5} &{} \dots &{} f_{K+2} \\ \vdots &{} \vdots &{} \vdots &{} \ddots &{} \vdots \\ f_{L} &{} f_{L+1} &{} f_{L+2} &{} \dots &{} f_{N} \end{bmatrix} \; , \; \left\{ \begin{array}{l} 1< L < K \\ K = N-L+1 \end{array} \right. \quad \end{aligned}$$

The matrix $\mathbf{X}$ is a symmetric matrix having identical elements on anti-diagonals. In the next step, we apply a singular value decomposition (SVD) to the trajectory matrix $\mathbf{X}$, i.e.,

9 $$\begin{aligned} \mathbf{X = U} \pmb {\varSigma } \mathbf{V}^\mathrm{T} \end{aligned}$$

with the superscript $^\mathrm{T}$ the transpose operator. The matrices $\mathbf{U}$ and $\mathbf{V}$ are orthonormal and are called left and right singular vectors, respectively. $\pmb {\varSigma }$ is a diagonal matrix containing nonnegative entries, the singular values, which reflect the importance of the singular vectors.

If $\lambda _1 \ge \lambda _2 \ge \cdots \ge \lambda _L \ge 0$ denote diagonal entries of $\pmb {\varSigma }$, the trajectory matrix can be written as:

10 $$\begin{aligned} \left\{ \begin{array}{l} \mathbf{X}=\mathbf{X}_1+\mathbf{X}_2+\cdots +\mathbf{X}_d \\ \mathbf{X}_i=\lambda _i \mathbf{U}_i \mathbf{V}_i^\mathrm{T} \end{array} \right. \end{aligned}$$

where $\lambda _i$ is the corresponding singular value of $\mathbf{X}_i$ and *d* is the number of nonzero singular values ($d \le L$).

In the next step, the grouping step, we select a group from $\{ \mathbf{X}_1, \mathbf{X}_2, \ldots , \mathbf{X}_d \}$ which will be used for reconstructing a new version of the time series. For instance, for reproducing a representative trend of the time series, we would choose the most critical part of $\mathbf{X}$ which is retrievable using the first few singular vectors and their corresponding singular values.

The last step is dedicated to the reconstruction of the time series using the selected group. Based on the fact that the original trajectory matrix had the same entries on anti-diagonals, we reconstruct a model of the time series ($G = (g_1,g_2,\ldots ,g_N)$) by anti-diagonal averaging:

11 $$\begin{aligned} g_i = \; \left\{ \begin{array}{ll} \frac{1}{i} {\sum \limits _{m=1}^{i}} \hat{x}_{m,i-m+1}&{} \; \; 1 \le i< L \\ \frac{1}{L} {\sum \limits _{m=1}^{L}} \hat{x}_{m,i-m+1} &{} \; L \le i \le K \\ \frac{1}{N-i+1} {\sum \limits _{m=i-K+1}^{N-K+1}} \hat{x}_{m,i-m+1} &{} K < i \le N \end{array} \right. \end{aligned}$$

where $\hat{x}_{i,j}$ is an estimation of the element $f_{i+j-1}$ of the original time series.
